# Hydrocortisone decreases metacognitive efficiency independent of perceived stress

**DOI:** 10.1038/s41598-020-71061-3

**Published:** 2020-08-24

**Authors:** Gabriel Reyes, Anastassia Vivanco-Carlevari, Franco Medina, Carolina Manosalva, Vincent de Gardelle, Jérôme Sackur, Jaime R. Silva

**Affiliations:** 1 Facultad de Psicología, Universidad del Desarrollo (UDD), Av. La Plaza 700, Las Condes, Santiago, Chile; 2 Institute of Pharmacy, Faculty of Sciences, Universidad Austral de Chile (UACh), Valdivia, Chile; 3 Paris School of Economics and CNRS, Paris, France; 4 Laboratoire de Sciences Cognitives Et Psycholinguistique (EHESS/CNRS/ENS), PSL Research University, École Normale Supérieure, 29 rue d’Ulm, 75005 Paris, France; 5 École Polytechnique, Palaiseau, France; 6 Clínica Alemana de Santiago, Santiago, Chile; 7 Instituto Milenio para la Investigación en Depresión y Personalidad (MIDAP), Santiago, Chile

**Keywords:** Human behaviour, Neurophysiology

## Abstract

It is well established that acute stress produces negative effects on high level cognitive functions. However, these effects could be due to the physiological components of the stress response (among which cortisol secretion is prominent), to its psychological concomitants (the thoughts generated by the stressor) or to any combination of those. Our study shows for the first time that the typical cortisol response to stress is sufficient to impair metacognition, that is the ability to monitor one’s own performance in a task. In a pharmacological protocol, we administered either 20 mg hydrocortisone or placebo to 46 male participants, and measured their subjective perception of stress, their performance in a perceptual task, and their metacognitive ability. We found that hydrocortisone selectively impaired metacognitive ability, without affecting task performance or creating a subjective state of stress. In other words, the single physiological response of stress produces a net effect on metacognition. These results inform our basic understanding of the physiological bases of metacognition. They are also relevant for applied or clinical research about situations involving stress, anxiety, depression, or simply cortisol use.

## Introduction

The negative impact of stress on human higher-order cognition is now well documented in cognitive neuroscience^1^. In particular, acute stress alters executive functions engaging the prefrontal cortex^2^, such as decision-making^3–5^, attention^6^, working memory^7–9^, learning^10,11^ or cognitive flexibility^12^. However, stress may affect cognition via multiple channels, which remain to be disentangled. At the physiological level, it is now well known that stress leads to a cascade of neuromodulator production, all of which impact brain functions, with a fast release of catecholamines (noradrenaline, dopamine, and then adrenaline) and a slower cortisol response very specific to stress^13,14^. These endocrine changes prepare the body to “fight or flight”, affecting breathing, heart rate, blood pressure, but also reducing prefrontal control, while enhancing amygdala function^2,13^. Another channel must be considered though, by which stress affects cognition via psychological factors, associated with how individuals perceive the situation and how they react to it (e.g., rumination^15^). Therefore, to evaluate the net effects of the biological components of stress (e.g. cortisol) on cognition, one should separate them from the psychological effects associated with the stress induction paradigm.

In this study, we aim at doing so by using a pharmacological approach. Within higher order cognitive functions, we focus on metacognition. This process refers to the ability to assess one’s own mental states^16^, for instance by evaluating the confidence that our own decisions are correct. Literature in this field has mainly focused on the cognitive determinants and neural bases of metacognition^17^, pointing to the key role of prefrontal cortex^18,19^. Two recent studies suggest that acute stress would impact metacognition. One study from our own group showed that individuals exhibiting a stronger cortisol response to social stress also presented poorer metacognition^20^, and a subsequent pharmacological study showed that metacognition improved following noradrenaline blockade^21^. Yet, the direct and causal effect of cortisol (which is the biological signature of stress) on metacognition remains unknown. Thus, in the present study we administered synthetic cortisol (hydrocortisone) to human volunteers to evaluate its effects on metacognition, in a single-blind hydrocortisone/placebo protocol. Importantly, by doing so we could induce a hormonal change similar to that created by social or physical stressors, without creating differences in the subjective experience of the situation or the subjective perception of stress, as controlled with behavioral evaluations. In other words, we could isolate the net biological effect of cortisol on metacognition. In this study we did not directly manipulate the stress of individuals, therefore our results should be mainly attributed to cortisol manipulation, a fundamental aspect of the stress response. We predicted that participants who received hydrocortisone would exhibit a lower metacognitive efficiency compared to controls.

## Material and methods

### Participants

Forty-six men (hydrocortisone group, *n* = 22; placebo group, *n* = 24; Mean (*M*) age = 24.3 years, Standard Deviation (*SD*) = 4.22) with a normal body mass index (*M* = 24.6, *SD* = 3.4) participated in the study. Individuals with psychiatric, cardiovascular, or metabolic disorders (abdominal circumference > 94 cm; capillary glycemia > 100 mg/dL [fasting] or > 200 mg/dL [post-meal]); alcohol or drug abuse (including tobacco); allergies; or hormonal treatment were excluded. Given the reported effects of oral contraceptives in exogenous cortisol administration^22^ and in HPA-axis activity^23^, we decided to exclude women from our study. Participants provided written, informed consent, and the guidelines of the Code of Ethics of the World Medical Association were completely fulfilled (Declaration of Helsinki). The ethics committees of Universidad del Desarrollo and Universidad Austral de Chile approved this study. All participants received $20 USD as compensation. Participants were informed that the aim of the experiment comprised evaluating the speed of visual discrimination, and that the potential effects of the drug, which had no harmful physical or psychological effect, would be reported at the end of the experiment.

### Stimuli and procedures

#### Questionnaires

**Figure 1:**
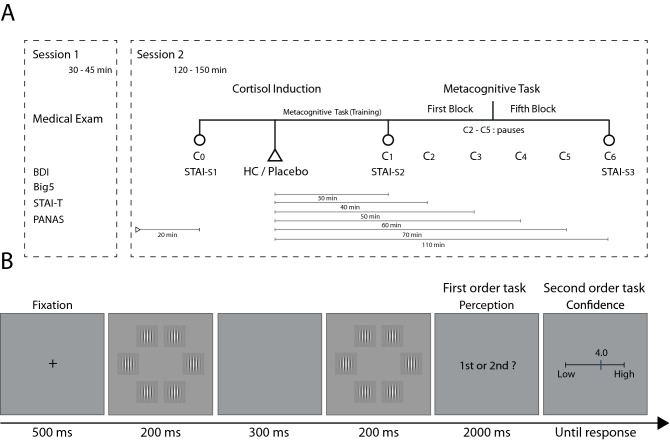
(**A**) Session 1 Personality trait questionnaires were assessed on an online platform. For Session 2, the pharmacological induction (placebo / hydrocortisone) was administered prior to the metacognitive task. Seven saliva samples and three STAI-S questionnaires were implemented. C0 and the first STAI-S were baseline measures, taken 20 min after the participant arrived at the lab. C1 and the second STAI-S assessment were sampled 30 min after drug administration, C2 to C5 were taken in when the task was paused, and C6 with the third STAI-S questionnaire was measured 30 min after the end of the metacognitive task. (**B**) Metacognitive task structure.

Several questionnaires were administered (before the experimental session) to obtain psychological profiles of the participants (Supplementary Material I, SM I). The level of depressive symptomatology was evaluated using the Beck Depression Inventory (BDI^24^); the tendency to experience positive and negative emotions was evaluated, using the Positive and Negative Affect Scale (PANAS^25^); participants’ anxiety personality traits were evaluated using the State-Trait Anxiety Inventory (STAI^26^) and the Big Five Personality Inventory (Big-5^27^). Preliminary results demonstrate that there are no significant differences in any of these questionnaires between the two experimental groups (for further detail please refer to Table SM-I). During the experimental session, three STAI-State questionnaires were implemented to evaluate the subjective consequence of the pharmacological induction, if any (Fig. 1-A).

#### Cortisol induction

Half of the participants received 20 mg of hydrocortisone and half received a placebo dose through an oral administration in a single-blind format. There are previous studies reporting an effect on cognition in response to ~ 20 mg hydrocortisone (10 mg [working memory]^28^; 10 mg [Response Inhibition]^29^; 20 mg [declarative memory]^30^; 20 mg [Inhibition]^31^; 30 mg [Declarative memory]^32^). Also, the described dose has significant effects on cortisol production as reported in independent studies^30,31,33^. As an example, Buchanan & Lovallo^30^ suggest that 20 mg of hydrocortisone generates comparable effects on cortisol production, as those evidenced in the context of real acute stress situations (e.g. military training^34^). Each hydrocortisone capsule was packed with polyvidone, sodium starch glycolate, microcrystalline cellulose, lactose monohydrate and magnesium stearate (Figure SM-IV for visual reference of the drug treatment). Participants received the drug with 250 ml of water to be swallowed immediately. After drug administration, participants began the training phase of the metacognitive task. This allowed the hydrocortisone dose to react and reach its hormonal peak at the beginning of the experimental task (~ 30 min^35^). Cortisol measures were obtained from saliva samples and immediately stored at -18 °C (SM II-A, for cortisol concentration analyses details). The first saliva sample was taken before administration of the drug (C0). Six samples were obtained, one at the start of the experiment and then during the pauses between blocks: (C1) 30 min after the cortisol induction, (C2) 40 min after, (C3) 50 min after, (C4) 60 min after, (C5) 70 min after, and (C6) 110 min after (Fig. 1-A).

### Experimental task

Stimuli were arrays of six vertical Gabor patches (2.8° in diameter, spatial frequency of 2.2 cycles per visual degree) on a uniform grey background (luminance: 44 cd/m^2^), presented on an imaginary circle (6.2°) at the center of a cathode ray tube screen (size 17" resolution of 1,024 × 768 pixels, refresh rate of 100 Hz, viewing distance ~ 55 cm). Participants performed this task in a darkened room. After a fixation spot (500 ms), participants viewed two arrays for 200 ms each, separated by an interval of 300 ms. In one of the arrays, one random Gabor patch had higher contrast. Participants had to decide at which interval the contrasted Gabor was presented, during a 2000 ms response window, by pressing the “Q” (first interval) or “W” (second interval) key on a standard QWERTY keyboard. During the experiment, contrast varied on a trial-by-trial basis, according to 1-up 2-down staircase method with the aim of adjusting the individuals’ performance to 71%^36^. Immediately after participants’ response, they were instructed to give an estimate of their confidence about their decision by means of a visual analog scale (range from 50 to 100 in intervals of 1). The experimental session comprised 300 trials in 5 blocks with ~ 1 min pause between each block, and 50 training trials (Fig. 1-B).

### Statistical analysis

We fitted our data using Linear Mixed Models (LMMs) using R (*lme4* package) and report the results of ANOVA tests on the fitted models. All our LMMs included fixed effects of Group (Placebo *vs*. Hydrocortisone group), of Time (From C0 to C6, both linear and quadratic), and a random intercept for participants. Following previous studies^18^, trials faster than 200 ms and slower than 2000 ms were excluded (exclusion of 2.4% of trials). For salivary cortisol measures, a linear and quadratic effects were analyzed based on the visual inspection of the data and considering the quadratic pattern of the cortisol scores with respect to other psychological functions reported in the literature (e.g., Lupien et al., 1997^1^ on Brain Cognition). Regarding metacognition analyses, it is necessary to ensure that metacognitive effects did not come from differences in first-order performance. Maniscalco and Lau^36^ proposed an unbiased signal detection theoretic approach^37,38^ to quantify metacognitive accuracy (meta-*d’*). It is then possible to capture the signal that is available for the second-order, metacognitive (type-2) task, and compare it against the type-1 (*d’*) task (i.e., meta-*d’*/*d’*: m-Ratio metacognitive efficiency index). Thus, meta-*d’* refers to the sensory evidence available for metacognition in signal-to-noise ratio units, just as type-1 *d’* is the sensory evidence available for decision-making in signal-to-noise ratio units. Therefore, meta-*d’*/*d’*, the m-Ratio, can be conceptualized as the individual metacognitive efficiency given a certain level of individual task performance^39^_._

## Results

### Salivary cortisol

**Figure 2:**
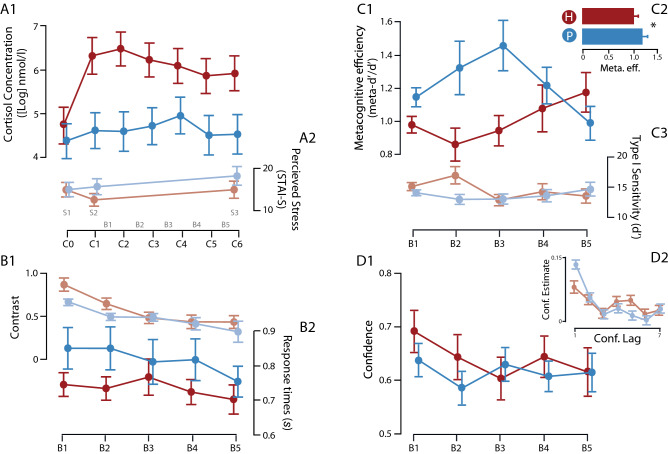
Comparison between Placebo and Hydrocortisone groups. The different panels represent (**A1**) cortisol concentration in saliva samples (power transformed40), (**A2**) perceived stress (as measured by the STAI-S), (**B1**) stimulus contrast, (**B2**) response times (in seconds), (**C1**) the metacognitive efficiency in the perceptual task (md-Ratio), (**C2**) type 1 sensitivity in the perceptual task (in d’ units), (**D1**) mean confidence (on a half-scale from .5 to 1) and (**D2**) confidence estimates in a serial dependency analysis. On each panel, dots and error bars represent the mean and standard error across participants, separately for Placebo group (in blue) and the Hydrocortisone group (in red). Labels (**C0**–**C6**) correspond to the cortisol samples, with (**C0**) being the baseline. Labels (**B1**–**B5**) correspond to the experimental blocks.

We first confirmed that cortisol levels (7 samples: from C0 to C6) was higher in the treated group. Note that in these analyses the cortisol scores were power transformed following previous recommendation^40^ (see SM II-B). Our linear mixed model on cortisol indicated a main effect of Group (*F*(62.98) = 6.81, *p* = 0.011), a quadratic effect of Time (*F*(272) = 17.69, *p* < 0.001), together with a significant interaction between these two effects (*F*(272) = 4.76, *p* = 0.030; Fig. 2-A1). Separate LMMs for the two groups indicated that the quadratic effect of time was present in the Hydrocortisone group (*F*(130) = 14.39, *p* < 0.001) but not in the Placebo group (*F*(142) = 3.19, *p* = 0.076). These main effects and the interaction maintain their significance after controlling for each personality trait questionnaire (all *p*s > 0.07). Regarding baseline cortisol levels, *t*-tests confirmed that salivary cortisol in the two groups was similar (*t*(43.5) = 0.616, *p* = 0.54), but diverged after the treatment between the two groups (average across C1-C5, Hydrocortisone *vs*. Placebo: *t*(43.66) = 2.80, *p* = 0.008). In short, our administration of Hydrocortisone *vs*. Placebo to participants had a clear impact on salivary cortisol, as expected. Finally, two indicators related to hormone levels were calculated: total amount of cortisol (Area under the curve with respect to the ground, AUCg) and total variation of cortisol during the experimental protocol (AUC with respect to the increments, AUCi; see SM II-C). Results evidenced that cortisol levels were greater in the Hydrocortisone than in the Placebo group (Fig. SM-II-C). Differences in BMI were explored across Groups (Hydrocortisone vs. Placebo). Results showed non-significant differences (p = 0.30) between these two groups. In a similar line, BMI is not significantly correlated with any Cortisol measures used in this study (C0-C6, AUCg, nor AUCi; all *p*s > 0.36). These results confirmed that pharmacological induction generates a differential effect on cortisol levels.

### Perceived stress

Subjective perception of stress was evaluated through the STAI-S questionnaire, which measures state anxiety. Our LMM indicated only a quadratic effect of Time (*F*(88) = 5.15,* p* = 0.026; Fig. 2-A2), with no other main effects or interactions (all *p*s > 0.05). In other words, perceived stress may have varied during the experimental protocol, but participants in the Hydrocortisone and Placebo groups did not have a different experience in terms of stress, suggesting that our protocol successfully induced a “biological stress” (as measured by cortisol) in the absence of psychological stress awareness.

### Task performance

In separate LMMs, we evaluated whether perceptual sensitivity, response time or stimulus contrast differed across the Hydrocortisone and Placebo groups. For stimulus contrast, there was a linear and quadratic effect of Time (*F*(177.7) = 84.5, *p* < 0.001; *F*(177.1) = 6.81, *p* = 0.010; Fig. 2-B1) but no effect or interaction with Group. For median Response Times (RTs), only a linear effect of Time was found (*F*(177.2) = 8.99, *p* = 0.003; Fig. 2-B2), again with no main effect or interaction from the Group factor. Perceptual sensitivity was affected by Group (*F*(217.4) = 6.86, *p* < 0.009) and by the interaction between Group and the linear effect of Time (*F*(178.32) = 4.37, *p* = 0.038; Fig. 2-C2). Separate *t*-tests within each block indicated that the Hydrocortisone and Placebo groups only differed in the second block (*t*(32.6) = 2.66, *p* = 0.012), and not in the other blocks (all *p*s > 0.13). Thus, except for a temporary increase in perceptual sensitivity, hydrocortisone administration has not affected task performance.

### Metacognition

Metacognitive efficiency was quantified using the m-Ratio measure. We evaluated whether metacognition differed between the Hydrocortisone and Placebo groups, while controlling for the possible influence of psychological stress, personality trait or task performance, by including in our LMM the following covariates: Perceptual Sensitivity (*d’* in each block), Response Times (median RT in each block), Contrast (average contrast in each block), Perceived Stress (average score across the 3 STAI-S questionnaires), and lastly all personality questionnaires (BDI, Stai-T, Big 5 and PANAS). This analysis revealed a main effect of Group (*F*(190.8) = 11.5, *p* < 0.001; Fig. 2-C3), to the effect that metacognitive efficiency was lower in the Hydrocortisone group, a main effect of Perceptual Sensitivity (*F*(203.7) = 23.4, *p* < 0.001), and an interaction between Group and the quadratic effect of Time (*F*(166.0) = 6.24, *p* = 0.013; Fig. 2-C1). To examine this interaction, we conducted separate LMMs for each Group. In the Hydrocortisone group, metacognitive efficiency increased linearly with Time (*F*(86.9) = 12.0, *p* < 0.001), whereas in the Placebo group it exhibited a quadratic effect of Time (*F*(93.5) = 4.44, *p* = 0.038). In addition, amongst our covariates, Perceptual Sensitivity was a significant predictor of metacognitive efficiency in both Groups (*F*(82.6) = 9.6 and *F*(111.7) = 28.8 in the Hydrocortisone and Placebo group, both *p*s < 0.005), as was stimulus contrast for the Hydrocortisone group (*F*(67.5) = 16.1, *p* < 0.001).

Finally, we evaluated the difference between the Hydrocortisone and Placebo groups separately within each block, again while simultaneously controlling for all aforementioned covariates using linear models. We found that the two groups differed significantly in all first 3 blocks (all *p* < 0.05) but not in the last two experimental blocks (both *p* > 0.3). In sum, in comparison to the Placebo group, the Hydrocortisone group exhibited a reduced metacognitive efficiency in the first 3 experimental blocks, even after controlling for task performance and self-assessment of stress.

### Confidence

For completeness, we examined the average confidence reported by observers in the two groups, in an LMM analysis in which we included the same covariates as above. We found no effect of Group, and no interaction of group with Time or with any of the covariates (all *p* > 0.2; Fig. 2-D1). Confidence was affected by Stimulus Contrast (*F*(190.2) = 15.4, *p* < 0.001), median RTs (*F*(213.5) = 9.27, *p* = 0.003), and Perceptual Sensitivity (*F*(179.7) = 16.9, *p* < 0.001), as could be expected from the literature on confidence in perceptual decision tasks. Finally, we then investigated the serial dependence of confidence judgments, relative to their confidence judgments in previous trials (from lag-1 to lag-7) for both conditions. In line with that reported by Rahnev et al.^41^, we performed a mixed regression analysis predicting confidence with fixed effects for the recent trial history up to seven trials back, for each condition, and random intercepts for each participant. We found evidence for strong autocorrelation in confidence, both in the experimental condition (lag-1[hydrocortisone]: *β* = 9.17e−02, *t*(5.952e + 03) = 7.080, *p* = 1.60e−12) and in the control condition (lag-1[placebo]: *β* = 1.50e−01, *t*(7.007e + 03) = 12.623, *p* = 2e−10), without a significant interaction (*p* = 0.21; Fig. 2-D2). These results suggest the existence of serial dependency in confidence, independent of the experimental condition.

### Questionnaires

Finally, to ensure that the two groups (Hydrocortisone *vs*. Placebo) did not differ in their psychological profiles, we used standard personality questionnaires (Table SM-I for analysis). Independent *t*-tests did not indicate any difference between the two groups in any of our questionnaires: BDI (*p* = 0.90), PANAS (positive affect: *p* = 0.66; negative affect: *p* = 0.14), STAI-T (*p* = 0.19), Big-5 (all *p*s > 0.13, Big-5 dimension independently evaluated). In other words, our treatment effect is not likely to be confounded with differences in personality traits between the two groups.

## Discussion

In this article, we investigated if metacognition could be modulated by a synthetic cortisol induction mimicking one crucial component of the physiological response to stress. The results confirmed our a priori hypothesis that metacognitive efficiency would be lower in the hydrocortisone condition than among controls. Our effects were not explained by differences in first-order performance (i.e., RTs, error rate, or *d*’) by considering one metacognitive score per subject, on a block by block basis, nor by taking into account cortisol increases from individual baseline (Cf., AUCi, SM-II-C). Furthermore, these effects are not explained by the subjective experience of participants: the hydrocortisone group did not report perceived stress during the experimental protocol and showed no differences on a number of psychological scales (i.e., STAI-T, BDI, PANAS, or Big-5). In this respect, one limitation of our study is that it is single blind. However, Although the experimenter knew participants’ assignments, the experimenter did not partake in the design of the study and had no knowledge of the hypothesis tested. Furthermore, in previous studies by other groups (see for instance Buchanan et al.^35^ on Pharmacology Biochemical Behavior) with the same hydrocortisone dosage and analogous setting, participants reported no conscious effect of hydrocortisone. Lastly, we found that treatment (hydrocortisone or placebo) had no impact on the State Trait Anxiety Inventory (STAI). We reasoned that if participants do not feel any psychological effect of the treatment, *a fortiori*, it is implausible that they should have some knowledge of their assignment. For all these reasons, we believe that it is unlikely that participants’ expectations might have been influenced by the experimenter's knowledge of the treatment condition, and that these might have influenced their metacognition in the task. Despite this, we show that cortisol per se had a negative impact on metacognition.

Our results suggest a dissociation between perceived stress and metacognition, as only the latter was affected by hydrocortisone administration: while participants in the Hydrocortisone group had poorer metacognition, they did not have access to this decrement, or at least it did not contribute to increase their perceived stress. In our study, metacognitive efficiency is affected by cortisol without creating a stressful experience for participants. While this has the methodological benefit that it isolates the activation of the cortisol pathway as sufficient in itself to the impairment of metacognition, we acknowledge that it raises the theoretically important issue of the nesting of the levels of metacognition, from perceptual metacognition to global emotional metacognition—an issue that our study is not well designed to approach.

Our hypothesis for an effect of stress and cortisol on metacognition stemmed from a previous study, in which we had shown that the cortisol response to a psycho-social stressor was negatively correlated with metacognitive sensitivity across individuals^20^. Critically, this previous study was only correlational, and consequently, it left two unanswered questions: First, it was unclear whether acute stress was the cause of the metacognitive efficiency drop, or whether a common underlying factor explained both responsiveness to stressors and poor metacognition; Second, even in the former alternative, it would still remain unclear whether stress affected metacognition via psychological factors or hormonal changes. The present study therefore fills a gap between stress, cortisol and metacognition, as it shows that cortisol increase is sufficient to hinder metacognition. We note nonetheless that a closer look at Fig. 2 reveals that the time course of the cortisol response and that of the metacognition modulation are not perfectly aligned. First, the effect of hydrocortisone intake on metacognition is postponed compared to its effect on cortisol concentration, with a delay of about fifteen minutes, which remains to be explained. Second, the difference in cortisol levels between the treated and placebo groups remained high in blocks 4 and 5, which showed however no difference in terms of metacognition, or even a reversal of the effect. The complex dynamics of the influence of cortisol on the brain^42^ might explain this return to baseline. Indeed, our latter blocks occurred approximately one hour after cortisol administration, a duration which has previously been associated with positive effects of cortisol on executive functioning, presumably because of the slow buildup of genomic effects of cortisol in the brain^43^. Admittedly, however, the detailed dynamics of the cortisol effect on metacognition remains to be investigated further. We note that the time course of metacognition in typical laboratory settings has not been, to our knowledge, investigated in detail. It might prove important to study the interaction of fatigue and training on first order performance and metacognition, as the inverted U-shaped curve that we observe for the m-Ratio in the Placebo group might be explained by their differential time course. As for the increase in metacognitive efficiency in the Hydrocortisone group, our interpretation remains even more speculative as it is unclear whether it should be theoretically expected that hydrocortisone administration should counteract fatigue effects or improve training. Further studies are clearly needed so as to clarify the time course of the impact of cortisol discharge on higher order cognition.

Stress triggers a complex cascade of biological responses, in which cortisol is but one of the components. Another component is the enhanced production of catecholamines (noradrenaline, dopamine, adrenaline). It is now widely thought that brain-wide noradrenergic activation creates a shift from reflexive, flexible processing (the defining hallmark of metacognition) to habitual, externally oriented processing^2,44–46^. Accordingly, a recent study^21^, showed that noradrenaline blockade via propranolol administration enhanced metacognition, by which one may speculate that stress could also degrade metacognition via the noradrenaline response—a finding which is coherent with the tight link between confidence estimation and pupil reactivity^47^. Yet, further research is needed to confirm this pathway, which might prove difficult. First, one should induce pharmacologically a noradrenaline increase that would mimic the typical short-lived noradrenaline response to stress. Second, one should ensure that this pharmacological manipulation does not affect the subjective perception of stress, which might otherwise constitute a confounding factor.

As much as noradrenergic blockade increases metacognitive efficiency^21^, we show that increased cortisol levels reduce metacognitive efficiency. We envision two main hypotheses with respect to the neural pathway of this effect: First, cortisol might have a *direct* down regulating effect on prefrontal areas subserving metacognition in perceptual tasks. Indeed, the literature about the neuroscience of metacognition^48^ and the neural effects of hypothalamic–pituitary–adrenal (HPA) activation^2^, converges on the notion that increased cortisol levels could impact metacognitive sensitivity through activation of Glucocorticoids Receptors (GR) located in the medial Prefrontal Cortex (mPFC). A major role of GR in mPFC is to generate a negative feedback loop for down regulating HPA axis activation^49^. Therefore, we speculate that this kind of signals may have a higher priority compared to those linked to metacognitive functions, diminishing their effectiveness. Second, cortisol levels might have an *indirect* effect on metacognition through interactions with noradrenergic activity, as was suggested for activity in the amygdala in response to emotional stimuli^50^. Further research could investigate this hypothesis, for instance by examining the joint effect of cortisol induction and noradrenergic blockade on metacognition. This would contribute to the weighting of the direct and indirect hypothesis for the impact of stress on metacognition, and by extension, to the understanding of the mechanisms of stress on higher level cognition.

Additionally, these results may contribute to clarify the debate regarding the nature of metacognitive ability. It remains controversial whether metacognition relies on a domain-general resource that is applied to different tasks or if self-evaluative processes are domain specific^51–54^. In this context, future studies interested in exogenously modulating metacognitive capacity (e.g., pharmacologically modulation^21^) could shed light on the nature (domain specific vs. general) of metacognition. From the domain-general thesis, it is possible that the cortisol effect here described denotes a massive effect on the neuronal circuitry that supports metacognition, such an effect should be independent of the type of sensory modality or cognitive task implemented. However, the neurocognitive architecture supporting metacognition remains controversial^51^. Fleming et al.^55^, for example, found evidence that individuals with anterior prefrontal cortex lesions, which performed two metacognitive tasks in two different domains (memory vs. perception), showed only a deficit in perceptual metacognition. In this vein, Ye et al.^56^ investigate the role of the medial parietal cortex (precuneus) in the metacognitive capacity of individuals in memory tasks and visual perception. Interestingly, the disruption of activity through transcranial magnetic stimulation (TMS) only negatively impacts metacognitive efficiency on memory tasks. Furthermore, the individuals' correlation in metacognitive scores in memory and perception disappears once the precuneus is perturbed. From our results, it is possible that the massive cortisol effect on metacognitive efficiency is related to a disturbance in the medial parietal cortex and associated cortical areas. Indeed, there is evidence regarding the negative effect on activity in precuneus related to the induction of cortisol^57^. Nevertheless, the hormonal effect evidenced here arises in the context of perceptual domain task; such cortisol effect could also consider an impact on frontal areas that integrate information from medial parietal cortex and amygdala to the medial PFC. Undoubtedly, specifying the neuro-hormonal effect of cortisol in metacognition will allow us to understand whether metacognition relies on mechanisms that are general or domain-specific.

In conclusion, we evidenced a direct and ecologically relevant pharmacological effect of cortisol on metacognition, that is, how individuals access their own mental contents. These results advance our understanding of the role of the underlying hormonal state on metacognitive capacity, about which very little is known (but see Hauser et al.^21^). The typical stress response implicates other hormonal changes and psychological dimensions, which could also impact metacognition. Our results demonstrate that independently of any of these, a cortisol increase in the range produced by typical psychosocial stressors is sufficient to alter metacognition in a perceptual task. Thus, our results suggest a new pathway for the detrimental effect of stress in decision making^1,4^, which could have a clinical impact on the subjective experience of stress and anxiety disorders^58,59^.

## Supplementary information

Supplementary file1.

## Data Availability

Dataset is available at the Open Science Framework at: https://osf.io/g6xnf/?view_only=9d8f7a87f6f940f88db44395a91025d2.
