# Supplementary material for: Hydrocortisone decreases metacognitive efficiency independent of perceived stress
Source: Sci Rep. 2020 Aug 24;10:14100. doi: 10.1038/s41598-020-71061-3 (PMC7445749; doi:10.1038/s41598-020-71061-3)
Supplement: Supplementary file 1 — Supplementary file1. [file 41598_2020_71061_MOESM1_ESM.docx]

**Supplementary Material:** Hydrocortisone decreases Metacognitive Efficiency independent of Perceived Stress

Gabriel Reyes, Anastassia Vivanco-Carlevari, Franco Medina, Carolina Manosalva, Vincent de Gardelle, Jérôme Sackur, & Jaime R. Silva

*Supplementary Material I: Questionnaires Description*

*Beck Depression Inventory* (BDI^1^). The BDI is a 21-item, self-reported questionnaire that evaluates the presence of depressive symptoms. The items ask for the frequency with which individuals have experienced different symptoms. Responses are made using a Likert-type response format with a range from 0 (*never*) to 3 (*always*). Total scores range from 0 to 63. This instrument has shown good psychometric properties among Chilean samples, similar to other Latin American samples^2^.

*Positive and Negative Affect Scale* (PANAS^3^). The PANAS is used to assess general emotionality. It comprises 20 items about how the individual usually feels in a series of feelings and emotions. They are scored on a Likert-type scale ranging from 1 (*very little or nothing at all*) to 5 (*extremely often*). Both positive and negative affect subscales have total scores ranging from 10 to 50. The PANAS is reliable and valid for Chilean samples^4^.

*State-Trait Anxiety Inventory* (STAI^5^). The STAI is used for reporting subjective stress states and traits, using two separate scales. State anxiety concerns temporary feelings of tension and apprehensive thoughts. Trait anxiety can be understood as the propensity of an individual to experience anxiety in any given stressful situation. Both scales comprise 20 items each, and they are answered using Likert-type responses ranging from 0 (*never*) to 3 (*very oft*en). Total scores in both scales range from 0 to 60. The STAI showed good internal consistency in a Chilean adult sample^6^.

*Big Five Inventory* (Big 5^7^). The Big 5 has 44 items. Participants respond to items using a Likert scale that reflects their level of agreement with a statement regarding their personality: from 1 (*strongly disagree*) to 5 (*strongly agree*). The personality dimensions are extraversion, agreeableness, conscientiousness, neuroticism, and openness. Total scores for each dimension range from 8 to 40. Previous studies have shown it has good internal consistency, and the five-dimension factorial structure has been replicated^8^.

===============

INSERT TABLE SM-I ABOUT HERE

===============

*Table SM-I* presents descriptive information of questionnaire scores across groups.


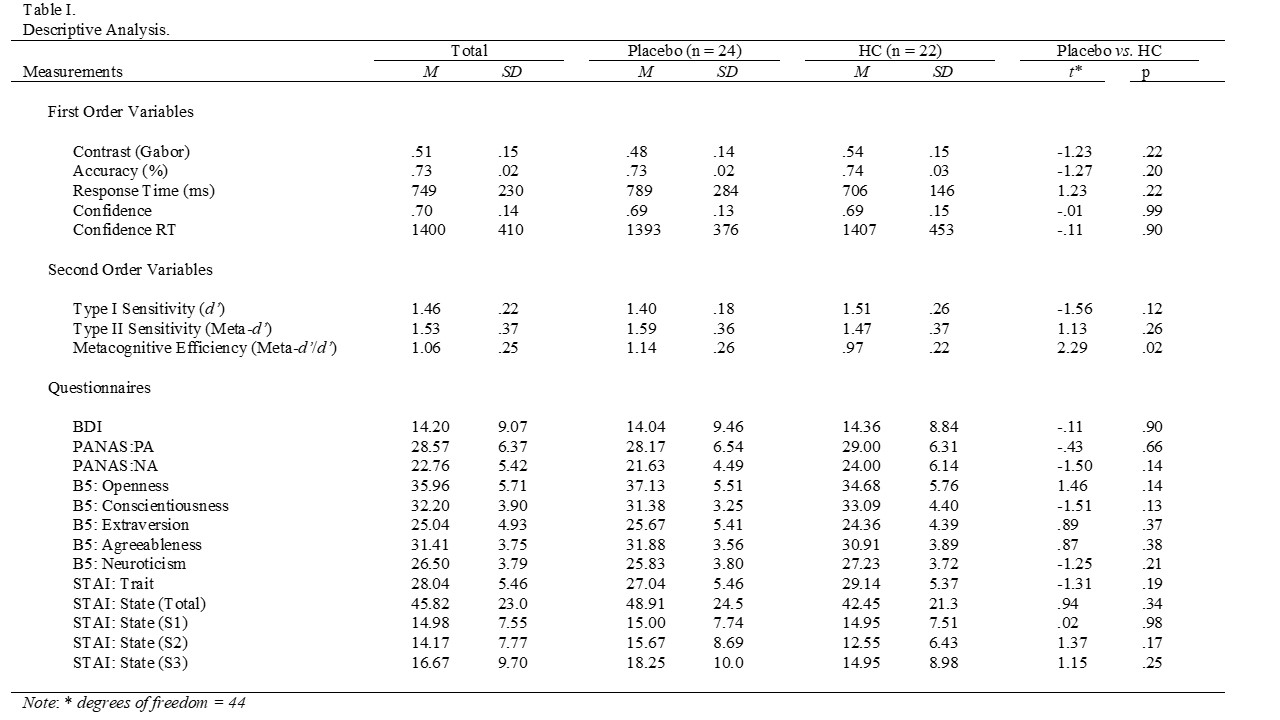


*Table SM-I.* Descriptive results (mean and standard deviation) of the whole sample and each group of the study (Placebo and Hydrocortisone). The last column presents the results of independent sample t-tests applied to evaluate differences across these two groups. The table contains results of first and second order measures of the visual task and scores of the different questionnaires applied on the protocol.

*Supplementary Material II-A: Cortisol Concentration Specification*

The saliva samples were thawed and centrifuged at 4000 g for 6 minutes; then, the determination of cortisol was conducted using a commercially available cortisol ELISA kit (R&D Systems, Inc., Minneapolis, MN, USA) according to the manufacturer’s specifications. Briefly, the ELISA technique (enzyme-linked immunosorbent assay), is based on the competition between the cortisol (antigen) and an enzyme-labeled antigen to bind with an antibody situated on the microplates. 100 μl of each sample were evaluated in duplicate, and the absorbance was measured in a microplate reader set to 450 nm (Stat Fax 2100, Awareness Technologies, MA, USA). The concentration of cortisol in each of the samples was obtained from the standard curve created in a concentration range between 0–10 ng/mL.

*Supplementary Material II-B: Cortisol Power Transformation*

Once the cortisol measurements were obtained along the seven samples, the distributions of concentrations of both groups were explored. Power transformations on individual measures were performed in order to normalize every distribution, following the methodological recommendations^9^ for temporary measures with stress induction:


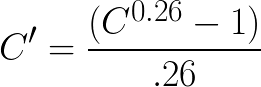


Where *C* is equal to the individual measure of cortisol and C’ represent the transformed cortisol value. Once transformed, normal distributions were obtained for the seven samples of saliva.

*Supplementary Material II-C: Cortisol AUC Analysis*

To confirm our experimental manipulation with respect to cortisol levels, two indicators related to hormone production were calculated: total amount of cortisol secreted (Area under the curve with respect to the ground, AUC_g_) and total variation of cortisol during the experimental protocol (AUC with respect to the increments, AUC_i_). For the calculation of both indicators, the formulae described in Pruessner, Kirschbaum, Meinlschmid & Hellhammer (2003)^10^ were used.

Regarding AUC_g_:


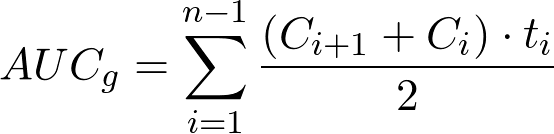


Regarding AUC_i_:


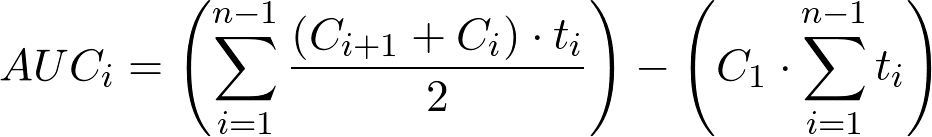


Where, for both formulae, *C_i_* correspond to a single measure of cortisol (Power transformed), *t_i_* denotes the time (in minutes) between each C, and *n* indicates the total number of cortisol samples taken during the protocol. We then performed two independent t-tests on AUC_g_ and AUC_i_ scores (both power transformed). Results indicated a higher production of cortisol for the Hydrocortisone group (*t*(44) = 2.61, *p* < .01, *η^2^_p_* = .13) in contrast to the Placebo group (Hydrocortisone: *M* = 591.8, *SD* = 148.7, *SE* = 31.7; Placebo: *M* = 458.54, *SD* = 192.5, *SE* = 39.3), and a higher overall increment of cortisol for Hydrocortisone group (*t*(31.4) = 2.17, *p* < .05, *η^2^_p_* = .09; Hydrocortisone: *M* = 118.4, *SD* = 191.7, *SE* = 40.8; Placebo: *M* = 20.8, *SD* = 102.4, *SE* = 20.9).

===============

INSERT FIGURE SM-II-C ABOUT HERE

===============


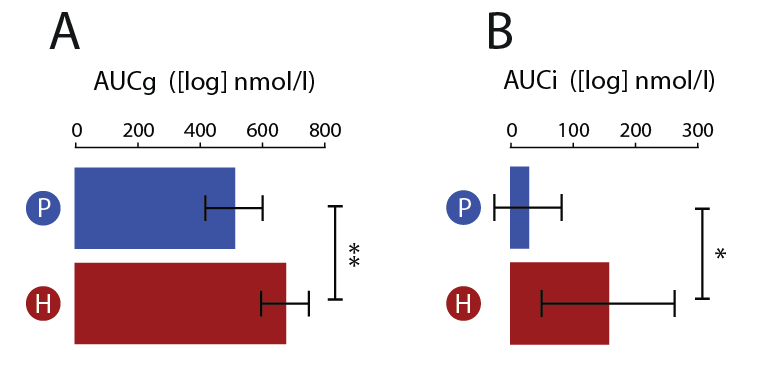


*Figure SM-II-C*. (A) Represent total amount of cortisol levels (area under the curve with respect to the ground, AUCg), and (B) total variation of cortisol during the experimental protocol (AUC with respect to the increments, AUCi), for each Condition (Hydrocortisone *vs*. Placebo). Error bars represent within-subjects 95% confidence intervals. * = *p* < .05; ** = *p* < .01; *** = *p* < .001; n.s.= non-significant.

*Supplementary Material III: Metacognitive Analysis*

Meta-*d’*/*d’* was obtained following Maniscalco and Lau’s (2012)^11^ methods, using maximum likelihood estimation. We used Matt Craddock’s R port of Maniscalco and Lau’s MATLAB functions ([https://github.com/craddm /metaSDT](https://github.com/craddm%20/metaSDT)), and we thank him for making these available to the community.

*Supplementary Material IV: Pharmacological treatment image*

===============

INSERT FIGURE SM-IV ABOUT HERE

===============


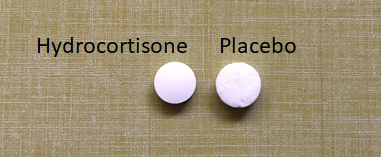


*Figure SM-IV:* Visual reference to the pharmacological treatment used in the experimental protocol. On the left the 20 mg dose of Hydrocortisone and on the right the 20 mg of placebo.

*Supplementary References*

1. Beck, A. T., Ward, C. H., Mendelson, M., Mock, J., Erbaugh, J. (1961) An inventory for measuring depression. *Arch. Gen. Psychiatry,* 4, 561-571 .

2. Valdés, C., Morales-Reyes, I., Pérez, J. C., Medellín, A., Rojas, G., Krause, M. (2017) Propiedades psicométricas del inventario de depresión de Beck IA para la población Chilena. *Rev. Méd. Chile* 145, 1005–1012.

3. Watson, D., Clark, L. A., Tellegen, A. (1988) Development and validation of brief measures of positive and negative affect: the PANAS scale. *J. Pers. Soc. Psychol.* *54,* 1063–1070.

4. Vera-Villarroel, P., Urzúa, A., Jaime, D., Contreras, D., Zych, I., Celis-Atenas, K., Silva, J. R., Lillo, S. (2017) Positive and Negative Affect Schedule (PANAS): psychometric properties and discriminative capacity in several Chilean samples. *Eval. Health Prof.* https://doi.org/10.1177/0163278717745344.

5. Spielberger, C. D., Gorsuch, R. L., Lushene, R. E. (1970) *The State-Trait Anxiety Inventory (Test Manual).* Consulting Psychologists Press.

6. Vera-Villarroel, P., Celis-Atenas, K., Cordova-Rubio, N., Buela-Casal, G., Spielberg, C. D. (2007) Preliminary Analysis and Normative Data of the State-Trait Anxiety Inventory (STAI) in Adolescent and Adults of Santiago. *Chile. Ter. Psicol.* *25*, 155–162.

7. John, O. P., Donahue, E. M., Kentle, R. L. (1991) *The Big Five Inventory-Versions 4a and 54*. University of California, Berkeley, Institute of Personality and Social Research.

8. Benet-Martínez, V., John, O. (1998) Los cinco grandes across cultures and ethnic groups: multitrait multimethod analyses of the big five in Spanish and English. *J. Pers. Soc. Psychol. 75*, 729–750.

9. Miller, R., Plessow, F. (2013) Transformation techniques for cross-sectional and longitudinal endocrine data: Application to salivary cortisol concentrations. *Psychoneuroendocrinol. 38,* 941-946.

10. Pruessner, J. C., Kirschbaum, C., Meinlschmid, G., Hellhammer, D. H., (2003). Two formulas for computation of the area under the curve represent measures of total hormone concentration versus time-dependent change. *Psychoneuroendocrinol. 28*, 916–931.

11. Maniscalco, B., Lau, H. (2012) A signal detection theoretic approach for estimating metacognitive sensitivity from confidence ratings. Conscious Cogn. 21, 422–430.
